# AMPK inhibition and elevated angiogenin are associated with tRNA fragmentation in the male germline exposed to a high-fat diet

**DOI:** 10.1016/j.molmet.2026.102350

**Published:** 2026-03-12

**Authors:** Eunbi Lee, Seo Yoon Choi, Seungmin Song, Anders M. Lindroth, Yoon Jung Park

**Affiliations:** 1 Department of Nutritional Science and Food Management, Ewha Womans University, Seodaemoon-gu, Seoul, 03760, Republic of Korea; 2 Graduate Program in System Health Science and Engineering, Ewha Womans University, Seodaemoon-gu, Seoul, 03760, Republic of Korea; 3 Graduate School of Cancer Science and Policy, Cancer Biomedical Science, National Cancer Center, Gyeonggi-do, Republic of Korea; 4 Global Food and Nutrition Research Institute, Ewha Womans University, Seodaemoon-gu, Seoul, 03760, Republic of Korea

**Keywords:** High-fat diet, tRNA-derived fragments, Angiogenin, Metabolic imprinting

## Abstract

The existence of an inherent rewiring of metabolic regulation has been demonstrated through studies of obese mice that are fed a normal diet and produce offspring with metabolic disorders. tRNA-derived fragments (tRFs) have been suggested as a potential mediator of this inheritance. To explore a mechanism underlying metabolic stress-induced tRNA fragmentation, we examined the effects of a HFD on tRFs in sperm. Small RNA sequencing on sperm revealed that HFD-induced metabolic changes affect the tRF profiles, showing a trend of decreased 5′-derived tRFs and increased 3′-derived tRFs, suggesting a shift in tRNA cleavage patterns under HFD feeding. In conjunction with the alteration of tRFs, the expression of *Ang*, which encodes the ribonuclease angiogenin, was significantly increased in the testis. Transcriptome and metabolome analyses indicated that AMPK-mTOR signaling pathway was the possible mediator of these effects, with decreased AMPK activity and increased mTOR activity confirmed at the protein level in the testis. Moreover, *in vitro* experiments showed that AMPK inhibition led to increased angiogenin expression level and alterations in tRF profile. *In vitro* angiogenin induction experiments, designed to mimic HFD conditions, produced changes in tRF profiles similar to those observed in HFD-fed mice, although the resulting tRF profiles did not completely recapitulate the *in vivo* HFD-induced profiles. Our findings suggest that HFD-induced metabolic stress inhibits AMPK during spermatogenesis, leading to increased *Ang* expression and altered tRF remodeling.

## Highlights

• Metabolic stress from a high-fat diet alters sperm tRNA-derived fragment profiles.
• HFD-induced metabolic stress shifts sperm tRF composition toward increased tRF3.
• AMPK regulates angiogenin expression through mTOR-independent mechanisms.
• Angiogenin induction under HFD conditions drives alteration of sperm tRF profiles.

## 1 Introduction

Obesity has become an epidemic, gradually increasing globally during the last 50 years [[1], [2], [3]]. Obesity is primarily caused by a prolonged positive energy balance, in which energy intake exceeds energy expenditure [4]. Especially, consumption of a high-fat or sucrose-rich diet contribute to excessive energy intake resulting in weight gain [5]. Weight gain significantly elevates risk of metabolic complications including cardiovascular disease, type 2 diabetes, and cancer [6,7].

Multiple evidences suggest that parents subject to environmental stress project physiological and metabolic traits to their offspring, potentially over several generations [8,9]. The phenomenon is referred to as intergenerational inheritance. Further studies have demonstrated that, similar to maternal inheritance, paternal diet-induced metabolic stress can lead to aberrant susceptibility of the offspring in subsequent generations [[10], [11], [12]]. In our previous study, we observed that obesity and metabolic stress caused by a high-fat diet (HFD) in male founders led to metabolic dysregulation in offspring over two generations, with sex-specific effects even when the offspring were fed a control diet (CD) [12]. Although this observation aligns with other independent reports, the specific mechanisms mediating this phenomenon have not been fully understood.

Epigenetic alterations in gametes, such as changes in DNA methylation or histone modification, and post-transcriptional regulation by miRNAs are frequently considered as leading contributors to the intergenerational inheritance [[12], [13], [14], [15], [16], [17], [18], [19], [20], [21]]. However, the specific alterations identified across studies rarely overlapped, raising concerns about whether these candidates can be considered reliable contributors [[12], [13], [14], [15], [16], [17], [18], [19], [20], [21]]. Several studies in recent years have identified tRNA-derived fragment (tRF), which is abundant in sperm, as a promising candidate of potent intergenerational mediators [[22], [23], [24]]. tRFs are post-transcriptional cleavage products of tRNAs, generated particularly in response to cellular stress by angiogenin, a ribonuclease belonging to the RNase A superfamily [[25], [26], [27]]. Studies from independent groups have shown that metabolic stress caused by HFD or low protein diet (LPD) leads to changes in the amount tRFs [22,23]. Zygotic injection of sperm-specific small RNAs, ranging from 30 to 40 nucleotides (nt) in size, which includes tRFs, from the HFD-fed mice resulted in metabolic dysregulation [22]. Notably, a one-week dietary intervention in human subjects led to alterations in tRFs [28], implying that tRFs sensitive to diet changes function as mediators of the inheritance of diet-induced metabolic stress from father to offspring.

Despite a plethora of studies linking intergenerational effects to diet-induced metabolic stress through male gametes that has suggested tRFs as promising candidate mediators of this phenomenon, major uncertainties of how diet-induced stress alters tRF remain. The objective of this study was to elucidate a potential mechanism underlying tRF alterations in response to HFD-induced metabolic stress in the male reproductive organ.

## 2 Methods

### 2.1 Animals

Animal experiments in this study were approved by the Institutional Animal Care and Use Committee of Ewha Womans University. Four-week-old C57BL6/J male mice were provided by the Ewha Laboratory Animal Genomic Center. These mice were divided into two groups and fed either a control diet (CD; 16% kcal from fat, AIN-93G, Saeronbio) or a high-fat diet (HFD; 60% kcal from fat, D12492, Saeronbio) for a period of 7 weeks (Table 1). All mice were maintained in specific pathogen-free conditions with a 12:12 h light–dark cycle and provided ad libitum access to water and food. Body weights and food intake were measured weekly, and all mice were sacrificed at 12 weeks for tissue sample collection. Blood samples were obtained through cardiac puncture from anesthetized mice. Following blood collection, whole brains were excised and weighed excluding the hypothalamus. Liver, kidney, inguinal white adipose tissue (iWAT), gonadal white adipose tissue (gWAT), and testis were also collected, weighed, and snap-frozen in liquid nitrogen.

**Table 1:** The composition of diets^a^.

	Control diet	High-fat diet
Ingredient (g/kg)		
Corn starch	397.49	–
Maltodextrin	132.00	125.00
Sucrose	100.00	72.80
Cellulose	50.00	50.00
Casein	200.00	200.00
l-cystine	3.00	3.00
Lard	–	245.00
Soybean oil	70.00	25.00
Mineral mix^a^	35.00	50.00
Vitamin mix^a^	10.00	1.00
Choline bitartrate	2.50	2.00
tert-Butylhydroquinone	0.01	–
Macronutrients (Calorie % of energy)		
Carbohydrate	63.9	20.1
Protein	20.3	20.0
Fat	15.8	59.9
Energy (kcal/g)	4.0	5.24

a Control diet (D10012G) and High-fat diet (D12492) were purchased from Research Diets, Inc. They were prepared with the mineral mix (S10022G and S10026B, respectively) and the vitamin mix (V10037 and V10001C, respectively).

Mature sperm samples were isolated from dissected epididymis, which were cut and incubated 15 min at room temperature with 500 μl of sterilized phosphate-buffered saline (PBS) to release the sperm. The PBS solutions containing sperm were gathered and subjected to centrifugation at 1,000 RCF for 15 min. The resulting pellets of sperm samples were rapidly frozen in liquid nitrogen. All tissues were stored at −80 °C prior to processing.

### 2.2 Chemicals and reagents

Compound C (CC, Abcam, Cambridge, UK; #ab120843), rapamycin (Sigma–Aldrich, Saint Louis, MO, USA; #37094), and MHY1485 (Sigma–Aldrich, Saint Louis, MO, USA; #SML0810) were used to modulate endogenous AMPK (AMP-activated protein kinase) and mTOR (mammalian target of rapamycin) activity. These compounds served as an AMPK inhibitor, mTOR inhibitor, and mTOR activator, respectively. Final concentration of 10 μM, 100 nM, and 2 μM were employed for CC, rapamycin, and MHY1485, respectively.

### 2.3 Cell culture

The mouse testicular TM3 cell line and TM4 Sertoli cell line were purchased from the Korean Cell Line Bank (Seoul, Korea). The NIH/3T3, TM3, and TM4 cell lines were cultured in Dulbecco's modified Eagle's medium (Welgene, Daegu, Korea; LM001-51) supplemented with 10% serum and antibiotics in a humidified atmosphere at 37 °C and 5% CO_2_ incubator. The specific serum for NIH/3T3 cell line was calf (Gibco, Gaithersburg, MD, USA; #26170-043), and fetal bovine serum (Corning, NY, USA; #35-015-CV) for the TM3 and TM4 cell lines.

### 2.4 RNA extraction, reverse transcription, and quantitative real-time PCR

RNA extraction from both cell lines and tissues was carried out using Trizol (Ambion, Carlsbad, CA, USA; #15596018). Subsequently, total RNA was reverse transcribed (RT) into complementary DNA (cDNA) using oligo dT and RevertAid Reverse Transcriptase (Thermo Fisher Scientific, Waltham, MA, USA; #EP0441) followed by *DNase*I digestion (Thermo Fisher Scientific, Waltham, MA, USA). Quantitative RT-PCR (qRT-PCR) was conducted with 2x QuantiNova SYBR Green PCR Master mix (QIAGEN, Hilden, Germany) on Rotor Gene Q (QIAGEN, Hilden, Germany) with gene-specific primers. The mRNA expression levels were normalized using primers to amplify the *Actin* or *Gapdh* genes. All primers used for qRT-PCR analysis are listed in Table 2.

**Table 2:** Primer sequences in this study.

Applications	Target		Primer Sequences (5’ – 3′)
qRT-PCR	*Actin*	F	ACTGCTCTGGCTCCTAGCAC
		R	ACATCTGCTGGAAGGTGGAC
	*Angiogenin*	F	CTCTGGCTCAGGATGACTCC
		R	CATCTTTGCAGGGTGAGGTT
	*Dnmt2*	F	TGCGATATTTCACACCGAAA
		R	CACATGCACGTTGAGGCTAT
	*Gapdh*	F	TGAAGGTCGGTGTGAACG
		R	CCATTCTCGGCCTTGACT
	*Nsun2*	F	F: ACTGTTGACCCAGGAGAACC
		R	R: TCTGGCTCATACTTCAGCACA
	*Tet2*	F	GCCAGAAGCAAGAAACCAAG
		R	CCTTCCTTCAGACCCAAACA
BT-tRNA (tRNA-Asp)	Stemloop-RT		CTCAACTGGTGTCGTGGAGTCGGCAATTCAGTTGAGTGGCTCCC
	Non-deaminated	F	TCCTCGTTAGTATAG
	Deaminated	F	AGTATAGTGGTGAGTATT
	Reverse	R	CACGACACCAGTTGA
Gateway	attB1		GGGGACAAGTTTGTACAAAAAAGCAGGCT
	attB2		GGGGACCACTTTGTACAAGAAAGCTGGGT
	*Angiogenin*-attB1		AAAAAGCAGGCTTCATGGCGATAAGCCCAGGCCCG
	*Angiogenin*-attB2		AGAAAGCTGGGTTCTATAGACTGAAAAACGACTC

### 2.5 Western blot analysis

Cell pellets from mouse testis and cell lines were lysed with RIPA lysis buffer (20 mM HEPES, pH 7.0, 150 mM NaCl, 10% glycerol, 1% Triton X-100, 1 mM EGTA, and 10 mM β-glycerophosphate). In the case of AMPK protein, cell pellets were lysed with AMPK lysis buffer (50 mM Tris–HCl, pH 7.4, 1 mM DTT, 1% Triton X-100, 250 mM Sucrose, 1 mM EGTA). Both buffers were supplemented with protease inhibitors cocktail (Roche, Basel, Switzerland; #11836153001), 1 mM PMSF, 1 mM Na_3_VO_4_, and 5 mM NaF. The protein concentration in each lysate was estimated with the Pierce BCA protein assay kit (Thermo Fisher Scientific, Waltham, MA, USA; #23225). Equal amounts of protein were subjected to electrophoresis on appropriate percentage SDS polyacrylamide gels. Subsequently, all gels were transferred onto PVDF membranes (Millipore, Burlington, MA, USA; #ISEQ00010). After the transfer, membranes were blocked with 5% skim milk or bovine serum albumin (BSA) in Tris-buffered saline with Tween-20 (TBS-T) for 1 h and then incubated with primary antibodies at 4 °C overnight. Signal detection was achieved using the LumiFlash Ultima Chemiluminescent substrate HRP system after incubation of peroxidase-conjugated secondary antibodies. Band density was quantified using Image J software and normalized against α-Tubulin or Actin. The antibodies used for Western blot analysis are listed in Table 3.

**Table 3:** Antibodies information.

	Antibodies	Company	Cat #	Dilution
Primary antibody	pAMPKα (Thr172)	Cell signaling technology	2535	1:1000 in BSA
	AMPKα	Cell signaling technology	2532	1:1000 in BSA
	pmTOR (Ser2448)	Cell signaling technology	5536	1:1000 in BSA
	mTOR	Cell signaling technology	2983	1:1000 in BSA
	α-Tubulin	Sigma aldrich	T5168	1:2000 in milk
	p4EBP1 (Thr37/46)	Cell signaling technology	2855	1:1000 in milk
Secondary antibody	Goat anti-rabbit IgG	Gene Tex	GTX213110-01	1:4000 in milk
	Mouse anti-rabbit IgG	Santa Curz	Sc-2357	1:4000 in milk
	Goat anti-mouse IgG	Rockland	610–1302	1:4000 in milk

### 2.6 Generation of overexpression vector

The Gateway technique was used to construct the overexpression plasmid. Full-length *Ang* coding sequence from tail gDNA obtained from C57BL/6N mice was amplified by PCR using Phusion High-Fidelity DNA polymerase (QIAGEN, Hilden, Germany). Following PCR, the product was purified with the QIAquick Gel extraction kit (QIAGEN, Hilden, Germany; #28704), and then subcloned into the pDONR221 donor vector through BP cloning reactions using Gateway BP Clonase 2 Enzyme mix (Invitrogen, Waltham, MA, USA; #11789-020). Recombinant clones were identified with RT-PCR using *Bsr*GⅠ (Thermo Fisher Scientific, Waltham, MA, USA; #ER0931), a restriction enzyme with a cutting site located on the recombinant site. Selected clones were subcloned into the destination vector, pDEST26 using the Gateway LR Clonase 2 Enzyme mix (Invitrogen, Waltham, MA, USA; #11791-020). Recombinant clones were identified with RT-PCR and *Bsr*GⅠ and finally confirmed by Sanger sequencing. All primer sequences are detailed in Table 2.

### 2.7 Small RNA sequencing of sperm and cell lines

Small RNA sequencing samples were prepared from sperm RNA sourced from CD and HFD mice, as well as CC-treated or non-treated TM4 cell lines, and TM4 cell line overexpressing *Ang*. Three sperm samples were pooled into one. Total RNA extraction was performed using Trizol, and 1 μg of total RNA was used for sequencing. Small RNA libraries were constructed following the TruSeq Small RNA Library Prep kit protocol from the manufacturer (Macrogen, Seoul, Korea). Read lengths of 140–145 nt, including adapters, were selected. Sequencing was performed on a NextSeq 500 platform, generating 37 to 64 million reads for each RNA library with 100 bp single-end reads.

Data quality was assessed using FastQC (v0.12.0). Reads were trimmed for TruSeq small RNA 3′ adaptor sequences (5′-TGGAATTCTCGGGTGCCAAGG-3′) and quality trimmed using Trimmomatic (v0.40). The trimmed reads were annotated using the SPORTS1.1 annotation pipeline with one mismatch and default settings. Reads ranging from 15 to 45 nucleotides were used and mapped to the small RNA database based on bowtie in the following sequence: rRNA database (NCBI), genomic tRNA database (GtRNAdb), mitochondrial tRNA database (mitoRNAdb), miRNA database (miRbase), non-coding database (Ensembl and Rfam), and PIWI-interacting RNA (piRNA) database (piRBase and piRNABank). Data files were obtained from the SPORTS1.1 website [29].

Total read counts, excluding rRNA-derived small RNA (rsRNA), were normalized based on the raw data size. Differentially expressed miRNAs and tRFs were analyzed using the DESeq2 R package, based on subclass in ‘summary.txt’ files. tRFs were normalized before using DESeq2 package. To normalize tRFs, reads mapped to the tRNA database were normalized by size factor, excluding rRNA, among all reads used as input for the SPORTS package. In the analysis of cell line data, batch effects were adjusted using the Combat-seq R package to reduce negative effects resulting from different technical processing during sample preparation.

### 2.8 Secondary analysis on publicly available metabolome data

To explore the metabolic changes induced by HFD feeding, a secondary analysis was conducted on publicly available metabolome data. Supplementary data obtained from a publication [30] by the Paolo Sassone-Corsi lab provided a comprehensive profile of metabolites across eight distinct tissues. Specifically, this study focused on the metabolite profiling of sperm. The metabolome data were collected at 4-hour intervals during zeitgeber time (ZT) for sperm samples (ZT0, 4, 8, 12, 16, 20). These samples were obtained from mice fed with either a CD or HFD of the same composition used in our study, with five samples per group.

Metabolites missing in more than half of the samples were filtered out. As a result, 142 metabolites in sperm were included in the subsequent analysis. Using the Generalized Linear Model (GLM) function in R version 4.1.1 using both variables (diet and ZT), significantly altered metabolites upon HFD feeding were identified. The significance criterion was set with a p-value threshold of less than 0.05 for the diet variable, leading to selection of metabolites that demonstrated significant alterations in response to HFD.

The significantly changed metabolites in sperm were used for pathway analysis. Metabolic pathway analysis was conducted using MetaboAnalyst (v5.0) with default settings (Enrichment method: hypergeometric test, Topology analysis: relative-between centrality) and the mouse KEGG library. Significantly changed pathways were selected based on a false discovery rate (FDR) < 0.05.

### 2.9 Northern blot analysis

For northern blots, 15 μg of RNA was used for electrophoresis on an 8 M 10% TBE urea gel, and the gels were subsequently transferred onto a positively charged nylon membrane (GE Healthcare, Amersham, UK; #RPN303B), followed by UV-crosslinking with an energy of 120 mJ. The membranes were pre-hybridized with Ultrahyb-oligo buffer (Thermo Fisher Scientific, Waltham, MA, USA; #8663) for 30 min, rotating in a hybridization oven at 42 °C. After pre-hybridization, the membranes were incubated with 20 pmol of ^32^P-labeled oligo probe in the same buffer, continuing from pre-hybridization to hybridization for 1 h in a rotating hybridization oven at 42 °C. After washing twice with wash buffer (2x SSC with 0.05% SDS) at 42 °C, each lasting 30 min, the membranes were exposed to autoradiographic film. Sequence of primers were: tRNA-Pro-AGG/CGG/TGG 5′-GGGCTCGTCCGGGATTTGAA-3’; tRNA-Val-AAC/CAC 5′-TGTTTCCGCCCGGTTTCGAA-3’.

### 2.10 Statistical analysis

Statistical analyses were performed using GraphPad Prism 9 and R version 4.1.1. Significance was assessed through Student's *t*-test or ANOVA, and values were expressed as means ± standard errors for *in vivo* data or means ± standard deviations for *in vitro* data. A p-value <0.05 was considered statistically significant.

## 3 Results

### 3.1 An assessment of the small RNA profile in sperm after diet interventions

**Figure 1:**
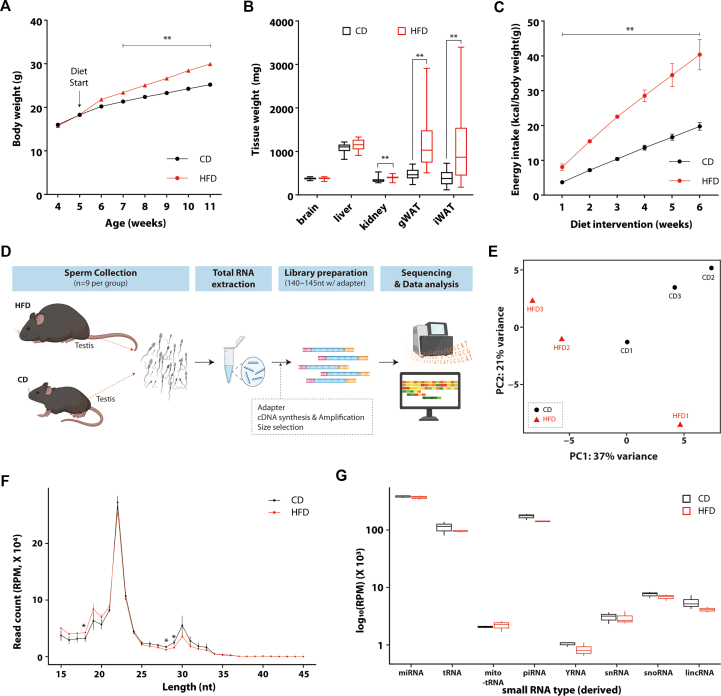
**Small RNA profiling of sperm is not much differed depending on diets. A** Line graph of body weight changes. Male mice fed CD and HFD from the 5 weeks of age. **B** Bar graph of tissue weights. Tissues were weighed at 12 weeks of age after sacrifice. **C** Cumulative energy intake during diet intervention. Data are presented as the means ± standard errors (CD, n = 20; HFD, n = 18). **D** Scheme of collecting sperm samples from nine mouse per group and small RNA sequencing. **E** PCA plot made with reads aligned using SPORTS. **F** Length distribution of reads used for small RNA sequencing analysis. RPM, Reads per million. **G** Read counts for individual small RNA type in both CD and HFD groups. Three pooled samples for each group, each containing sperm from three mice. Statistical analysis was performed using the student's *t*-test. ∗, *p* < 0.05 and ∗∗, *p* < 0.01 vs CD.

In order to examine HFD-induced changes in testis linked to intergenerational effects of paternal metabolic stress, male mice were fed with HFD for 7 weeks. The HFD group had higher diet-induced body weights than the CD group (Figure 1A). The weight difference was significant already after three weeks of the diet intervention. The weights of iWAT and gWAT were likewise increased in the HFD group (Fig. 1B). Additionally, the HFD group consumed considerably more energy than the CD group (Fig. 1C). In line with the present results, a previous independent set of animals fed the same HFD exhibited impaired glucose regulation based on an intraperitoneal glucose tolerance test (Supplementary Fig. 1).

Paternal gametes are set to carry only a limited set of molecules besides DNA across generations, primarily in the form of transcripts and a restricted repertoire of proteins [31]. We reasoned that ribonucleic acids were of prime importance in transmitting metabolic alterations, leading us to perform total and small RNA sequencing on the sperm from both CD and HFD mice. Initial analysis by total RNA sequencing revealed only minimal differences between sperm from CD and HFD mice (supplementary Fig. 2), which prompted further analysis with small RNA sequencing (Figure 1D,E). During library preparation for sequencing, size selection was performed to obtain fragments with a total length of 140–145 bp, including both the insert and adapter sequences. Sequencing reads were analyzed using the SPORTS1.1 pipeline, focusing on reads ranging from 15 to 45 nt in length. By analyzing the size distribution of fragments, there was no notable difference. In terms of small RNA length between the diet groups. However, HFD had a significantly lower read count within the size range of 28–29 nt, which mainly corresponds to piRNAs and tRFs, compared to CD (Fig. 1F). To assess the impact of HFD on the composition of small RNAs in sperm, we quantified the abundance of each small RNA subtype. However, HFD had no significant effect on change of small RNA proportion (Fig. 1G). Although there was no significant change in the proportion of each small RNA subtype, the reduced abundance of 28–29 nt reads under HFD is consistent with a trend toward decreased levels of tRFs and piRNA, which mainly fall within the size range. Collectively, these data indicated that HFD has not significantly changed the overall profile of small RNA in sperm.

### 3.2 HFD-induced metabolic changes are mirrored by altered tRFs in sperm

**Figure 2:**
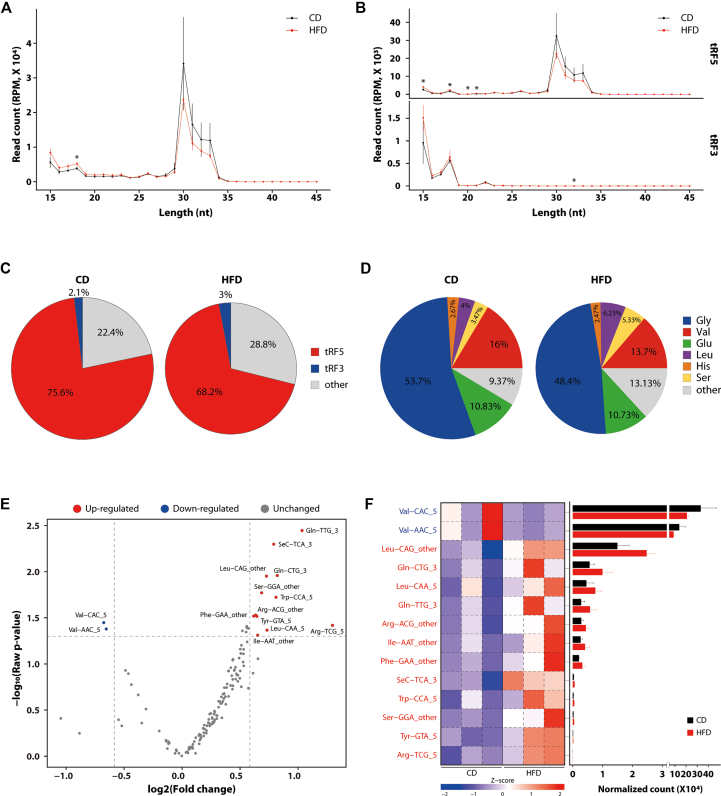
**tRF profiling of sperm is changed in response to HFD feeding. A** Length distribution of reads aligned to GtRNAdb. **B** Length distribution of reads annotated as tRF5 and tRF3 among aligned to GtRNAdb. **C** Distribution of reads assigned to GtRNAdb in tRF classes. tRF5, reads derived from tRNA 5′ end among reads aligned to GtRNAdb; tRF3, reads derived from tRNA 3′ end among reads aligned to GtRNAdb; other, not included in the previous two groups. **D** Distribution of reads assigned to GtRNAdb in tRNA family type. Gly, Glycine; Val, Valine; Glu, Glutamic acid; Leu, Leucine; His, Histidine; Ser, Serine; Other included alanine, arginine, asparagine, aspartic acid, cysteine, glutamine, isoleucine, lysine, methionine, phenylalanine, proline, selenocysteine, threonine, tryptophan, and tyrosine. Pie chart size is proportional to the total read count. **E** Volcano plot depicting differentially expressed tRFs in sperm between HFD versus CD. **F** (Left) Heatmap showing the significantly differentiated tRF relative expression levels based on Z-score transformed scale. (Right) Normalized counts of tRFs showing left heatmap. Statistical analysis in **A** and **B** was performed using the student's t-test. ∗, p < 0.05 vs CD.

Given that tRFs have been suggested as putative mediators of specific metabolic states [22,23,32], we analyzed the sperm small RNA sequencing data with a focus on tRFs aligned to GtRNAdb, a genomic tRNA sequence reference database. The most abundant reads in the CD group that matched the GtRNAdb were 30–35 nt in length that fell within the category of tRF5 (Figure 2A,B). In contrast, the HFD group had a higher number of reads under 20 nt (Fig. 2A), suggesting a shift toward shorter tRF species, which may reflect altered tRNA cleavage patterns associated with HFD-induced metabolic stress.

Structural analysis of tRFs indicated that the tRF5 fragments, derived from the 5′-end of tRNAs, were most prevalent in both groups, with the HFD group having lower levels than the CD group (Fig. 2C). Fragments emanating from tRNA sequences other than the 5′ or 3′ end were classified as “other”, and the proportion of these fragments increased in the HFD group. In line with this pattern, the level of tRF3 fragments, derived from the 3′-end of tRNAs, was relatively higher in the HFD group (Fig. 2C). A closer examination of the tRNA origins of the tRFs revealed that tRNA-Gly was the most prevalent species in both groups, followed by tRNA-Val and tRNA-Glu (Fig. 2D).

We then identified the tRFs whose levels had changed due to HFD feeding. When all tRF types were examined, 14 out of 148 showed significant changes in the HFD group compared to the CD group (Fig. 2E). Especially, the relative amount of tRF5 derived from tRNA-Arg-TCG and tRF3 derived from tRNA-Gln-TTG increased more than twofold, with statistically significant differences (Fig. 2E). The tRF5 derived from tRNA-Val was the only tRF that decreased in the HFD group, with the highest count, suggesting that this contributed to the overall reduction trend in tRF5 levels of the HFD group (Figure 2C,E,F). Our analysis of small RNA sequencing data using sperm suggested that HFD-induced metabolic changes can affect the profile of tRFs in the sperm, which could function as mediators transmitting metabolic information induced by diet from the paternal generation.

### 3.3 AMPK inhibition induces *Ang* expression in testis

**Figure 3:**
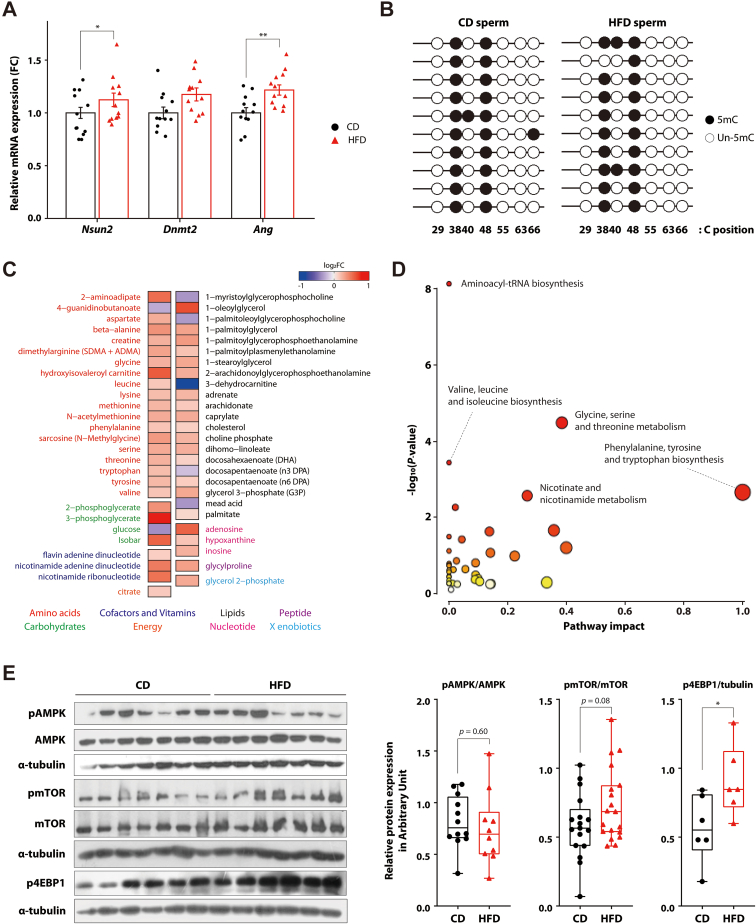
**AMPK inhibition is a candidate mechanism of HFD-induced Angiogenin induction. A** mRNA expression changes after HFD feeding in testis compared to CD. Relative levels of mRNA expression were calculated by normalization against Gapdh expression. Data are presented as means ± standard errors (CD, n = 20; HFD, n = 18). Statistical analysis was performed using the student's t-test. ∗, p < 0.05 and ∗∗, p < 0.01 vs CD. **B** Methylation status of tRNA-Asp-GTC in sperm by bisulfite analysis. Five clones per sample were used (n = 2 per group, total 10 clones per group). Black circles represent methylated CpG sites and white circles represent unmethylated CpG sites. **C** Heatmap showing significantly changed metabolites in sperm of mice fed CD and HFD. The red and blue color indicates relatively high and low fold change (HFD compared to CD), respectively. **D** Scatter plot showing metabolic pathway induced in sperm by HFD feeding. Pathway analysis was performed with MetaboAnalyst5.0. The node color indicates p-value, and the node radius indicates the pathway impact. Significant pathway names were labelled near the nodes. **E** (Left) The representative Western blot result of proteins related with AMPK-mTOR pathway. pmTOR/mTOR was measured 17 times in 10 biologically different samples in the CD group and 19 times in 8 biologically different samples in the HFD group. (Right) The graph of quantified the band intensities of Western blot results using ImageJ software. Data are presented as mean ± standard deviation.

To investigate how the profiles of tRFs are altered, we examined the expression levels of tRNA-modifying enzymes in the testis, including genes encoding the DNMT2 and NSUN2 tRNA methyltransferases, and angiogenin. The expression of the *Nsun2* and the *Ang* genes were significantly higher in the HFD group than in the CD group, while *Dnmt2* expression did not change in the testis (Figure 3A). These findings suggested the possibility that tRNA methylation status might have been differentially altered by increased expression of tRNA methylation-related genes rather than all tRNAs. To determine if HFD impacted the methylation status on tRNA, we performed bisulfite sequencing analysis of tRNA, in particular tRNA-Asp-GTC, which is a common target of DNMT2 and NSUN2 [[33], [34], [35]]. While the expression level of the *Nsun2* gene was increased, there were no differences in the methylation status of sperm tRNA-Asp-GTC at neither the cytosine 48 (C48) target site of NSUN2, nor the cytosine 38 (C38) target site of DNMT2 (Fig. 3B).

To better understand how the metabolic landscape influences tRF alterations, we used publicly available metabolome data emanating from identical diet intervention as our study [30]. To this end, we used sperm metabolome data from mice subjected to a HFD and found significant alterations in their metabolite composition. Out of the 142 sperm metabolites, 53 showed statistically significant differences between HFD and CD groups, with 46 metabolites were increased and 7 were decreased in the HFD group (Fig. 3C). Notably, there was a marked increase in lipid levels, particularly in various fatty acids such as docosapentaenoate (DPA), docosahexaenoate (DHA), arachidonate, palmitate, and adrenate, suggesting enhanced lipid accumulation in sperm under HFD conditions. Similarly, several amino acids – such as leucine, phenylalanine, valine, tyrosine, and serine – were also elevated in the HFD group, indicating that both energy-related and biosynthetic pathways may be affected by dietary fat intake.

We next conducted pathway analysis using 46 significantly induced metabolites by HFD feeding to discover significantly changed pathways in sperm. As a result, five significant pathways were obtained, all of which were involved in amino-acid metabolism, and the top induced pathway was aminoacyl-tRNA biosynthesis, which is strongly linked to the amino acid metabolism and mTOR signaling pathway (Fig. 3D). Transcriptomic analysis of the testis from HFD-fed mice showed only limited expression changes, however *Kit*, a gene previously implicated in RNA-mediated intergenerational inheritance, was notably downregulated (Supplementary Fig. 2a-c) [36]. In addition, downregulated genes are associated with thermogenesis and oxidative phosphorylation, suggesting altered energy expenditure in response to HFD feeding (Supplementary Fig. 2d, e) in line with the metabolome analysis. As the AMPK-mTOR signaling pathway is an essential element of the nutrient sensing system and a central regulator of protein synthesis, we investigated its role as a central mediator of this phenomenon [37]. This analysis showed that there was a trend, while not statistically significant, of decreased phosphorylation of AMPK and increased phosphorylation of mTOR in the HFD group compared to the CD (Fig. 3E). Consistent with the observed AMPK and mTOR changes, the p4EBP1, which acts downstream of mTOR, became significantly activated in the HFD group (Fig. 3E). These findings indicated a novel link in sperm between the AMPK-mTOR signaling pathway and HFD-related metabolites elevated by HFD.

### 3.4 AMPK inhibition alters tRFs profiles mediated by *Ang* induction

**Figure 4:**
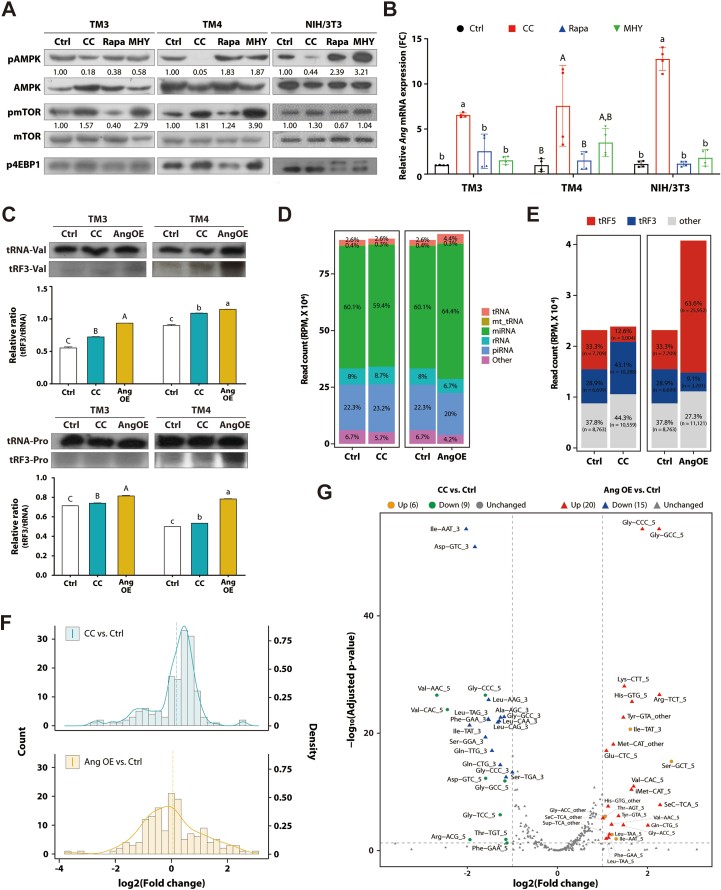
**AMPK inhibition-induced Angiogenin is accompanied by alteration of tRFs profile in vitro. A** Western blot results of proteins related with AMPK-mTOR pathways in TM3, TM4, and NIH/3T3 cell lines using compound C (CC), rapamycin (Rapa), and MHY1485 (MHY) in TM3, TM4, and NIH/3T3 cell lines (left to right). The number represents the densiometric comparison of the expression of pAMPK and pmTOR proteins against AMPK and mTOR, respectively. **B** Angiogenin gene expression. Gene expression was quantified after 12 h of treatment. mRNA expression level was normalized to *Actin*. Data are represented as mean ± standard deviation. Statistical analysis was performed using One-way ANOVA with Tukey post-hoc test. Different letters are significantly different. **C** Northern blot results of tRF3-Pro and Val in mouse testis cell lines. Each sample RNAs were extracted from both TM3 and TM4 cells with CC treatment and overexpression of Angiogenin (Ang OE). Each intact tRNA was shown as a control. Band intensities of Northern blot are represented as bar graph after quantification using Image J software. Data are presented as mean ± standard deviation. One-way ANOVA was used for statistical analysis, and the different alphabets with Tukey post-hoc test. **D** Distribution of read counts based on small RNA type. **E** Distribution of reads assigned to GtRNAdb in tRF subtypes. The total bar height is proportional to the total read count for each group. Percentages indicate the relative proportion of each tRF subtype within the corresponding group, and *n* represents the actual read count number for each subtype. tRF5, reads derived from tRNA 5′ end among reads aligned to GtRNAdb; tRF3, reads derived from tRNA 3′ end among reads aligned to GtRNAdb; other, not included in the previous two groups. **F** Histogram showing the distribution of log_2_(Fold change) using reads aligned to GtRNAdb. Green, CC-treated versus non-treated; Yellow, ANG overexpression versus control. **G** Volcano plot depicting significantly changed tRFs in TM4 cell lines. Results from CC-treated versus non-treated are represented as circles, and results from ANG overexpression versus control are represented as triangles. n = 2 for each group.

To further investigate the AMPK-mTOR's involvement in metabolic stress-induced upregulation of the *Ang* gene, we used the TM3 and TM4 mouse cell lines, isolated from prepubertal gonads and mouse fibroblast NIH/3T3 cell line. To manipulate endogenous AMPK-mTOR pathway signaling we treated the cell lines for 12 h with CC, rapamycin, and MHY1485. We confirmed with Western blot analysis that the chemicals affected the protein activity of AMPK and mTOR (Figure 4A). Notably, CC treatment, providing AMPK inhibition at the protein level, led to an increase in *Ang* expression (Fig. 4B). However, altering mTOR protein activity using rapamycin and MHY1485 as an antagonist and an agonist, respectively, did not significantly affect *Ang* expression (Figure 4A,B). As the impact of CC on *Ang* induction was consistent across all three cell lines, it did not appear to be cell-type specific (Fig. 4B). Furthermore, Northern blot analysis confirmed that CC treatment and *Ang* overexpression induced the tRNA fragmentation (Fig. 4C). Collectively, the results indicated that AMPK inhibition led to an increase in *Ang* expression, which in turn drives alterations in tRNA fragmentation.

To further explore whether inhibition of AMPK induced *Ang* expression and altered tRNA fragmentation, we performed small RNA sequencing analysis on CC treated- and *Ang* overexpressed OE-TM4 cells. The analysis of reads revealed that 2.6% of tRNAs matched the GtRNAdb in the control group and remained unchanged in the CC-treated group (Fig. 4D). However, there was a slight increase in expression of *Ang* OE tRNA from 2.6 to 4.4% (Fig. 4D), consistent with the notion that angiogenin contributes to generate tRFs [25,26]. When examining the distribution of tRFs by their origin, both CC treatment and *Ang* expression caused changes in the proportion of specific tRF specimens (Fig. 4E). We also observed that the overall abundance of individual tRFs increased under both conditions compared to the control group, with a more pronounced increase observed in the CC treatment (Fig. 4F). In an attempt to identify significantly altered tRFs in both conditions, our analysis showed that 6 tRFs were upregulated and 9 were downregulated in the CC-treated group, while 20 tRFs were upregulated and 15 were downregulated in the *Ang* induced group (Fig. 4G). These findings suggested that both CC treatment and *Ang* overexpression influenced tRF abundance, with each condition showing distinct changes. Several fragments – such as those derived from Ile-AAT (CC-treated group), Gln-CTG, Phe-GAA, Sec-TCA, and Tyr-GTA (Ang OE group) – were found to be upregulated under both CC/Ang OE and HFD conditions, despite differences in the origins of the tRFs (Figures 3E and 4G). Notably, tRF5 derived from tRNA-Val-ACC and tRNA-Val-CAC were consistently downregulated under both CC-treated and HFD groups. Collectively, these results suggested that while CC treatment and *Ang* induction did not fully recapitulate the tRF pattern altered by HFD, distinct changes in tRF profiles between *Ang* induction and AMPK inhibition correlated closely.

## 4 Discussion

In this study, our focus was on understanding the molecular context in which diet-induced metabolic stress is associated with tRNA fragmentation in the male germline. We first analyzed the changes of small RNA profiles in response to metabolic stress induced by HFD through RNA sequencing-based analysis. The tRFs, particularly the relatively shorter tRF3s, were increased in sperm from the HFD group compared to the CD group. HFD feeding affected expression of tRNA-modifying genes in testis, including those regulating methylation and cleavage. Although tRNA methylation analysis did not reveal significant differences at the specific nucleotide sites we examined, its fragmentation was induced upon HFD feeding, corresponding with the upregulation of angiogenin, a that accommodate its tRNA cleavage. The induction of angiogenin in testis due to HFD was mimicked by AMPK inhibition, but not mTOR modulation in testicular cell lines. AMPK-inhibited cell lines also showed changes of tRFs, especially induction of tRF3 amounts, although the overall distribution of tRFs were different from that observed in mature sperm. In summary, this study demonstrated how HFD-induced chronic metabolic stress led to AMPK inhibition and *Ang* overexpression in testis, accompanied by alteration of sperm tRFs.

Multiple pieces of evidence indicated that tRFs could serve as promising candidates for mediators of intergenerational effect [22,23,32,38]. In a study of human subjects, there were observed differences in sperm tRF profiles between lean and obese men [28]. Intriguingly, a short-term intervention with a high-sugar diet for just one week resulted in significant changes in the tRF profile in sperm [28]. Consistent with previous findings in mice, 5′ tRF were the most abundant species, whereas 3′ tRF were the least abundant [23,28]. In mouse models exposed to chronic LPD or HFD showed increases in 5′ tRF, particularly those derived from Gly-GCC, Gly-CCC and Glu-CTC tRNAs [22,23]. In the human study of short-term high-sugar diet, however, the tRF subtypes showing significant changes were more frequently internal tRFs (i-tRFs), originating from internal regions of mature tRNAs, as well as 3′ tRFs [28]. Notably, the specific 5’ tRF species reported to change in mouse models were not significantly affected in human sperm following the high-sugar diet [28]. The study hence suggested that tRFs in sperm are susceptible to the influence of diet stress. In our study, nevertheless, the overall amounts of tRFs did not exhibit a significant difference between HFD and CD groups, indicating that reads shorter than 22 nt increased, while those between 30 and 35 nt decreased in the HFD group (Fig. 2B). The variations in the total amounts of tRFs observed under metabolic stress have reported inconsistencies across various studies [22,23,32]. For instance, Chen et al. reported an increase in the total quantity of tRFs in the sperm of mice subjected to the HFD [22], while Sharma et al. observed no significant difference in the overall amount of tRFs [23]. In contrast, Zhang et al. reported a decrease in the total amount of tRFs in their supplementary data [38]. These discrepancies suggested that it may not be the total quantity of tRFs that is crucial for transmission of metabolic stress, but rather the quality that carries greater significance. Importantly, high-fat diet exposure represents a state of metabolic overload accompanied by mitochondrial dysfunction, altered cellular energetics, and chronic inflammation, rather than an acute cytotoxic stress response [39]. A recent report has demonstrated that mitochondrial stress produces a tRNA fragmentation profile distinct from canonical oxidative stress, characterized by heterogeneous and often shorter tRNA-derived fragments rather than dominant long tRNA halves [40]. This framework is consistent with our observation of modest enrichment of shorter fragments and supports the interpretation that metabolic state influences qualitative features of tRF populations. The HFD model is not intended to reproduce an ancestral dietary condition but to provide a controlled state of nutrient-driven metabolic imbalance for mechanistic study. Such responses may reflect evolutionarily conserved programs that once enabled adaptation to fluctuating food availability but appear maladaptive under chronic energy-rich environments.

Although several studies have been conducted on the function of tRF on translational regulation [[41], [42], [43]], ribosome synthesis [44,45], and cell differentiation [46,47], lack of studies examining how tRFs, which are altered by HFD, contribute to intergenerational effects after entering the zygote hamper mechanistic understanding. Chen et al. suggested that sperm tRFs could affect regulation of embryonic gene expression as early as the 8-cell stage in the embryo by sequence-matching on gene promoter regions after sperm entered the zygote [22]. At present, only bulk or size-selected sperm RNA populations have been experimentally linked to transmission phenotypes. Whether specific tRFs altered by HFD exert functional effects after fertilization remains unresolved. Beyond regulating other genes, tRFs also modulate the transcription of their parental tRNAs. For example, tRF5-Glu and -Gly were found to promote the transcription of their tRNA genes, thereby regulating embryonic development in zebrafish embryos [48]. Through this mechanism, gene expression and translation can be regulated by tRFs [45,49].

Previous studies have reported the generation tRFs from sperm in response to various metabolic stresses. For instance, HFD feeding induced levels of tRF5-Gly and tRF3-Gly, while LPD increased levels of tRF-Gly, Glu, Lys, and His, but decreased tRF-Phe and Arg [23,38]. Another study using inflammatory mouse model reported increased levels of tRF-Gly, iMet, Glu, Val, His, Arg, and Met in sperm [32]. In our analysis, HFD led to a slight increase in the amount of tRF3 and a decrease in the amount of tRF5 (Fig. 2C). At the amino acid level, tRF5-Arg, Tyr, Trp, and Leu, as well as tRF3-SeC and Gln were increased in the HFD group, whereas tRF5-Val was decreased (Fig. 2F). The patterns observed in AMPK-inhibited or Ang-induced cell lines did not fully recapitulate the *in vivo* findings (Fig. 4), likely reflecting differences in cellular context and experimental system. Such variability indicates that metabolic stress may influence RNA fragmentation processes in a context-dependent manner, with the final sperm RNA profile representing the integrated outcome of multiple biological layers rather than a single regulatory event. The reasons for variations in the quantity or proportion of specific tRFs across studies remain unclear, and whether certain tRFs play a crucial role in the paternal transmission of metabolic stress remains to be determined. Only total or size-selected small RNAs from sperm of mice fed with HFD and subjected to microinjection experiments have so far been directly demonstrated to contribute to paternal transmission [22,23,32]. To establish the importance of specific tRFs, microinjection experiments involving the injection of particular tRFs into fertilized zygotes would be necessary.

Intriguingly, microinjection of synthesized individual tRFs, estimated to represent 70% of the endogenous tRFs in sperm, did not reproduce the transmission of metabolic stress as effectively as did microinjection of endogenous tRFs [22]. This discrepancy might be attributed to the presence of methylation at cytosine (5 mC) and guanine (7 mG), which were notably enriched in tRFs from the HFD group [22]. This finding aligned with other studies indicating that tRNA methylation played a significant role in the process of tRNA fragmentation during metabolic stress [34,50]. The absence of the tRNA methyltransferase DNMT2 resulted in increased tRFs that did not function as mediators of the intergenerational effect [38]. Contrasting the findings by Chen et al. discussed above, an independent study demonstrated that zygotic injection of synthetic unmethylated tRFs partially mimicked the intergenerational effect of paternal inflammation-induced stress [32]. Our analysis of tRNA methylation revealed no difference between sperms from mice fed with HFD and CD (Supplementary Fig. 2b), suggesting that the fragmentation of tRNAs, rather than their methylation, might be a vital component in the metabolic stress response. However, we cannot rule out the possibility that methylation on other tRNAs might be crucial for paternal transmission, as our study only assessed methylation at mature forms of tRNA-Asp-GTC in sperm under diet stress [51].

The significance of this study rests on the induction of tRFs by metabolic stress, especially through the upregulation of *Ang* expression in the testis in response to the HFD feeding (Fig. 3A). Induction of angiogenin has previously been reported under ER (endoplasmic reticulum) stress conditions [52]. Although angiogenin is well known to generate canonical tRNA halves under acute stress conditions, recent evidence indicates that angiogenin activation does not necessarily dictate the final size distribution of tRNA-derived fragments [40]. Instead, angiogenin may function as an initiating nuclease whose effects are subsequently shaped by additional RNases, RNA turnover pathways, and metabolic state–dependent processing. This framework may explain why the HFD model preferentially showed enrichment of shorter and 3′-derived fragments despite *Ang* induction. Additionally, angiogenin activity is tightly regulated by its endogenous inhibitor, ribonuclease/angiogenin inhibitor 1 (RNH1). Under normal growth conditions, angiogenin is predominantly localized in the nucleus, where it supports rRNA biogenesis and protein synthesis, while cytosolic angiogenin is bound by RNH1, which suppresses its RNase activity [[53], [54], [55]]. Upon stress conditions, angiogenin translocates to the cytoplasm as RNH1 relocates in the opposite direction, enabling tRNA fragmentation [53]. Thus, dissociation of angiogenin from RNH1 is likely required for its catalytic activity [25]. Given that HFD induces inflammation and cellular stress, it is plausible that HFD-associated stress may promote the release of angiogenin from RNH1, thereby facilitating tRNA fragmentation.

Notably, a recent study indicated that the absence of angiogenin prevented the intergenerational transmission of inflammation-induced metabolic disorder [32]. Despite the extensive research on the stress response mediated by angiogenin [56], little is known about how metabolic stress induces its upregulation. To answer this question, our analysis of the sperm metabolome showed an upregulation of pathways related to aminoacyl-tRNA biosynthesis and various amino acid biosynthesis reactions in response to HFD feeding (Fig. 3D). These HFD-induced increases suggest enhanced protein production capacity, potentially influencing the rate of translation. Therefore, we focused on the AMPK-mTOR signaling pathway as an upstream regulator affecting the sperm metabolome, because AMPK-mTOR signaling pathway is a key component of the nutrient sensing system and serves as a central regulator of protein synthesis [37].

The transcriptional activation of the *Ang* gene was mimicked by AMPK inhibition. Initially, we used CC, an AMPK inhibitor, not only to repress AMPK but also to subsequently activate the mTOR pathway. Unexpectedly, the induction of *Ang* expression was not recapitulated by the mTOR activator MHY1485 (Fig. 4B). One possible explanation for the difference in results with the mTOR modulators CC and MHY1485, could be due to the mechanisms by which the mTOR complex are activated. CC activates mTOR complex by inhibiting AMPK and the mTOR inhibitory effect of the tuberous sclerosis protein complex (TSC) [57], while MHY1485 activates the mTOR complex by directly binding to mTOR protein [58]. Consistently, co-treatment with CC and Rapamycin still resulted in *Ang* induction (Supplementary Fig. 3), suggesting that this effect may occur through an mTOR-independent mechanism. This finding suggested that inhibition of AMPK, rather than a direct increase in mTOR, is required for *Ang* induction. In a d-galactose-induced renal aging model, hyperoside inhibits AMPK-ULK1-mediated autophagy without affecting mTOR, indicating an mTOR-independent mechanism [59]. Likewise, in the absence of intracellular Ca^2+^, autophagy is induced via an AMPK-dependent, mTOR-independent, mechanism [60]. The activation of mTOR by inhibiting AMPK was one of possibly many outcomes, as it cannot be ruled out that *Ang* expression could be induced through pathways distinct from mTOR. It is noteworthy that the critical point is that merely overexpressing angiogenin did not mimic the tRF pattern changes observed under HFD consumption in the results (Figure 4E), suggesting that the metabolic environment created by AMPK inhibition appears to modulate how tRNA cleavage products are processed and stabilized together with *Ang* induction.

In summary, our findings demonstrate that HFD-associated metabolic stress is accompanied by coordinated but modest changes in AMPK signaling, angiogenin expression, and the distribution of specific sperm tRF subsets. These results describe a stress-responsive RNA remodeling phenomenon rather than a single-enzyme–driven mechanism or a proven pathway of paternal transmission. Future studies incorporating epididymal biology and functional embryo assays will be required to determine whether and how these RNA changes contribute to offspring phenotypes.

## CRediT authorship contribution statement

**Eunbi Lee:** Writing – original draft, Visualization, Investigation, Formal analysis, Data curation. **Seo Yoon Choi:** Investigation. **Seungmin Song:** Investigation. **Anders M. Lindroth:** Writing – review & editing, Supervision, Funding acquisition. **Yoon Jung Park:** Writing – review & editing, Supervision, Project administration, Funding acquisition, Conceptualization.

## Funding

This work was supported by the 10.13039/501100003725National Research Foundation of Korea (NRF) grant funded by the Korea government (10.13039/501100014188MSIT) (RS-2025-00573031 to YJP) and National Cancer Center of Korea (2510450 to AML).

## Declaration of competing interest

The authors declare that they have no known competing financial interests or personal relationships that could have appeared to influence the work reported in this paper.

## Data Availability

Small RNA sequencing data generated in this study have been deposited in the Gene Expression Omnibus (GEO) under accession number GSE323392.
