# Supplementary material for: AMPK inhibition and elevated angiogenin are associated with tRNA fragmentation in the male germline exposed to a high-fat diet
Source: Mol Metab. 2026 Mar 12;107:102350. doi: 10.1016/j.molmet.2026.102350 (PMC13049662; doi:10.1016/j.molmet.2026.102350)
Supplement: Multimedia component 1 [file mmc1.docx]

**Supplementary Information**

**AMPK inhibition and elevated angiogenin are associated with tRNA fragmentation in the male germline exposed to a high-fat diet.**

Eunbi Lee, Seo Yoon Choi, Seung Min Song, Anders M. Lindroth, Yoon Jung Park

**Supplementary Methods**

**Supplementary Fig. 1** Impaired glucose tolerance of HFD-induced mice.

**Supplementary Fig. 2** Analysis of differentially expressed genes upon HFD feeding in testis transcriptome.

**Supplementary Fig. 3**

**Supplementary methods**

***Intraperitoneal glucose tolerance test (IPGTT)***

Following a 12-hour fast, 2g/kg of 25% glucose solution (Sigma, MO, USA) was injected into the intraperitoneal space to conduct the intraperitoneal glucose tolerance test. Accu-Check Performa (Roche, IN, USA) was used to measure blood glucose levels from tail at fasting (0) and 15, 30, 60, and 120 minutes after the injection.

***Total RNA sequencing of testis***

RNA sequencing analysis was conducted on 1 µg testis RNA obtained from both CD and HFD mice, with three samples per group utilized for sequencing. Total RNA was extracted using Trizol. Total RNA libraries were constructed using the TruSeq Stranded Total RNA Library Prep Gold Kit following the manufacturer (Macrogen, Seoul, Korea)’s protocol. Sequencing was performed on a NovaSeq 6000 platform, generating over 100 million reads per each RNA library with 100 bp paired-end reads.

To assess the raw data quality, FastQC (v0.12.0) was used, employing a phred quality score. Reads were aligned to the GRCm39 reference genome from GENCODE with a comprehensive gene annotation file using STAR (v2.7.10b). FeatureCounts (v2.0.6) was used to quantify reads in each sample, and DESeq2 R package (v3.17) calculated differentially expressed genes between the groups. Statistically differentially expressed genes (DEGs) were defined with an adjusted p-value < 0.05 and |fold change| > 1.5. DEGs were analyzed for GO-term enrichment via the Enrichr website (https://maayanlab.cloud/Enrichr).

***Bisulfite-based methylation analysis of sperm tRNA***

The RNA extracted from sperm using Trizol was subjected to electrophoresis on a 10% TBE-urea precast gel (Bio-rad Laboratories, Hercules, CA, USA; #4566033), and the gel was then stained with SYBR Green 1 (Thermo Fisher Scientific, Waltham, MA, USA; #S7563). Gel fragments ranging from 10 to 100 bp were selectively isolated and used in subsequent experiments. RNA in the excised gel was purified using acid phenol/chloroform and was subjected to bisulfite conversion through the EZ RNA methylation kit (Zymo Research, Irvine, CA, USA; #R5001). The bisulfite-treated RNA was reverse transcribed using SuperScriptⅡ RTase (Thermo Fisher Scientific, Waltham, MA, USA; #18064022) with a tRNA-Asp-GTC gene specific primer. HotstarTaq DNA polymerase (QIAGEN, Hilden, Germany; #203203) was used to amplify tRNA-Asp-GTC. The resulting PCR products were separated on a 2% agarose gel and purified using the QIAGEN Gel Extraction kit (QIAGEN, Hilden, Germany; #28704). After purification, the PCR products were subcloned with the TOPO TA cloning kit (Invitrogen, Waltham, MA, USA; #450641) and sequenced to verify the methylation status. The primes used for this analysis are listed in Table 2.

**
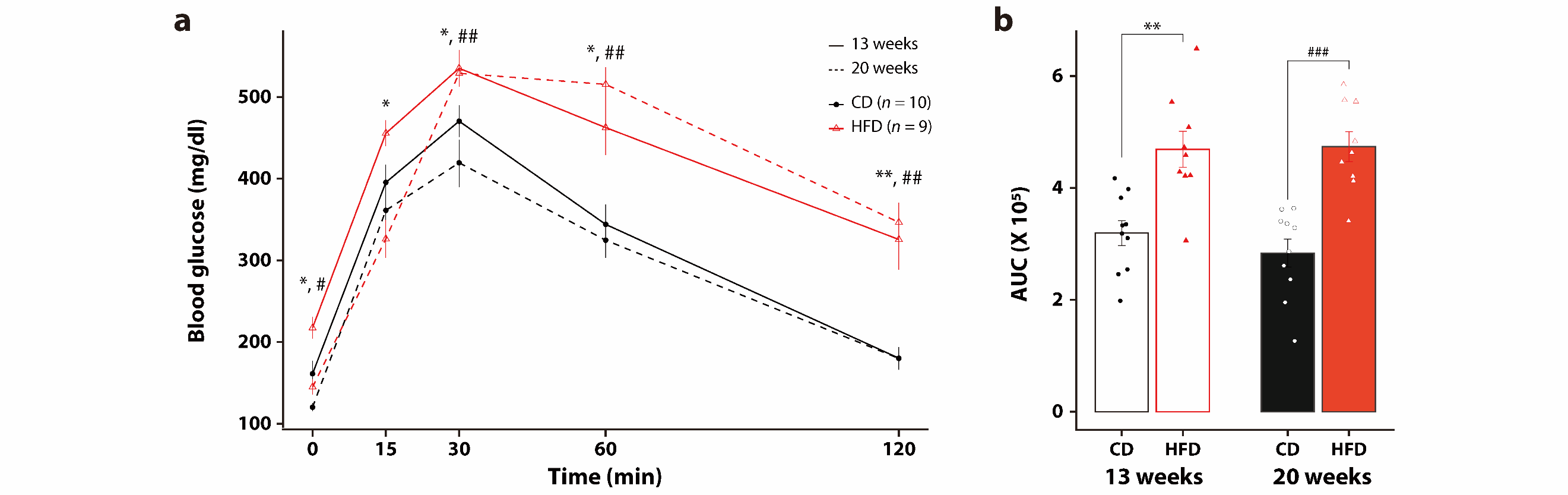
**

**Supplementary Figure 1. Impaired glucose tolerance of HFD-induced mice.**

Glucose levels upon intraperitoneal glucose tolerance tests at 13 and 20 weeks of age. Black and red lines indicate mice fed with CD and with HFD, respectively. Solid and dotted lines indicate results from 13 and 20 weeks of age, respectively. Data are presented as means ± standard errors (CD, n=10; HFD, n=9). Statistical analysis was performed using the student’s *t*-test, *, *p*<0.05 and **, *p*<0.01 vs CD.

**
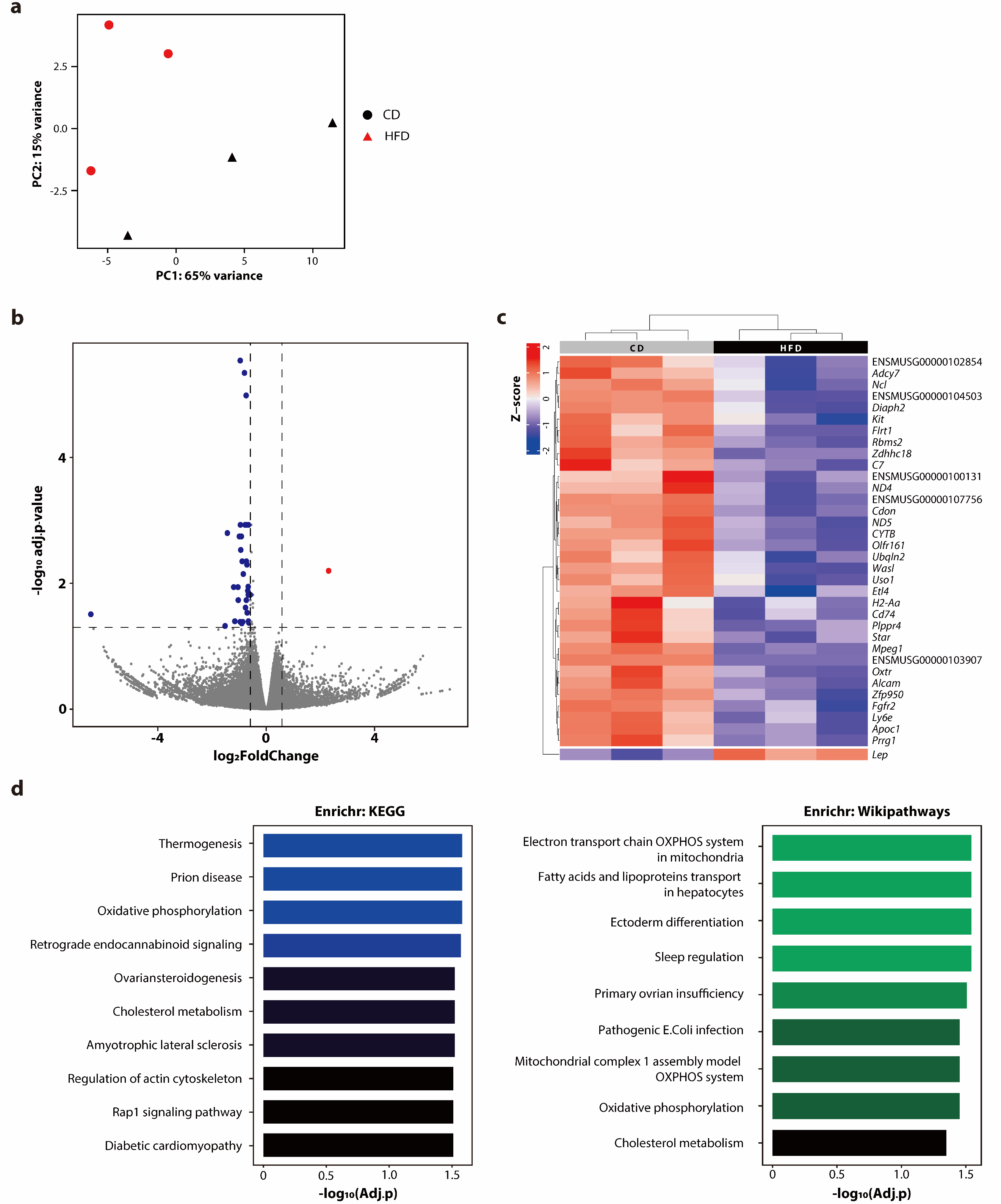
**

**Supplementary Figure 2. Analysis of differentially expressed genes upon HFD feeding in testis transcriptome.**

(a) PCA plot from testis transcriptome for diet variables. (b) Volcano plot generated for differentially expressed genes (DEG) between HFD and CD. The red dot indicates up-regulated gene, and the blue dots indicate down-regulated genes in the HFD group compared to the CD group. The gray dots denote the gene expression without marked differences. The threshold was set as |Fold Change|>1.5 and adjusted p<0.05. (c) The expression heatmap of all DEGs between HFD and CD with hierarchical clustering results. The red and blue color indicates relatively high and low z-score (HFD compared to CD), respectively. (d) Bar plots showing the top 10 KEGG pathway results on the left and top 9 WikiPathways on the right for downregulated genes in the HFD group.

**
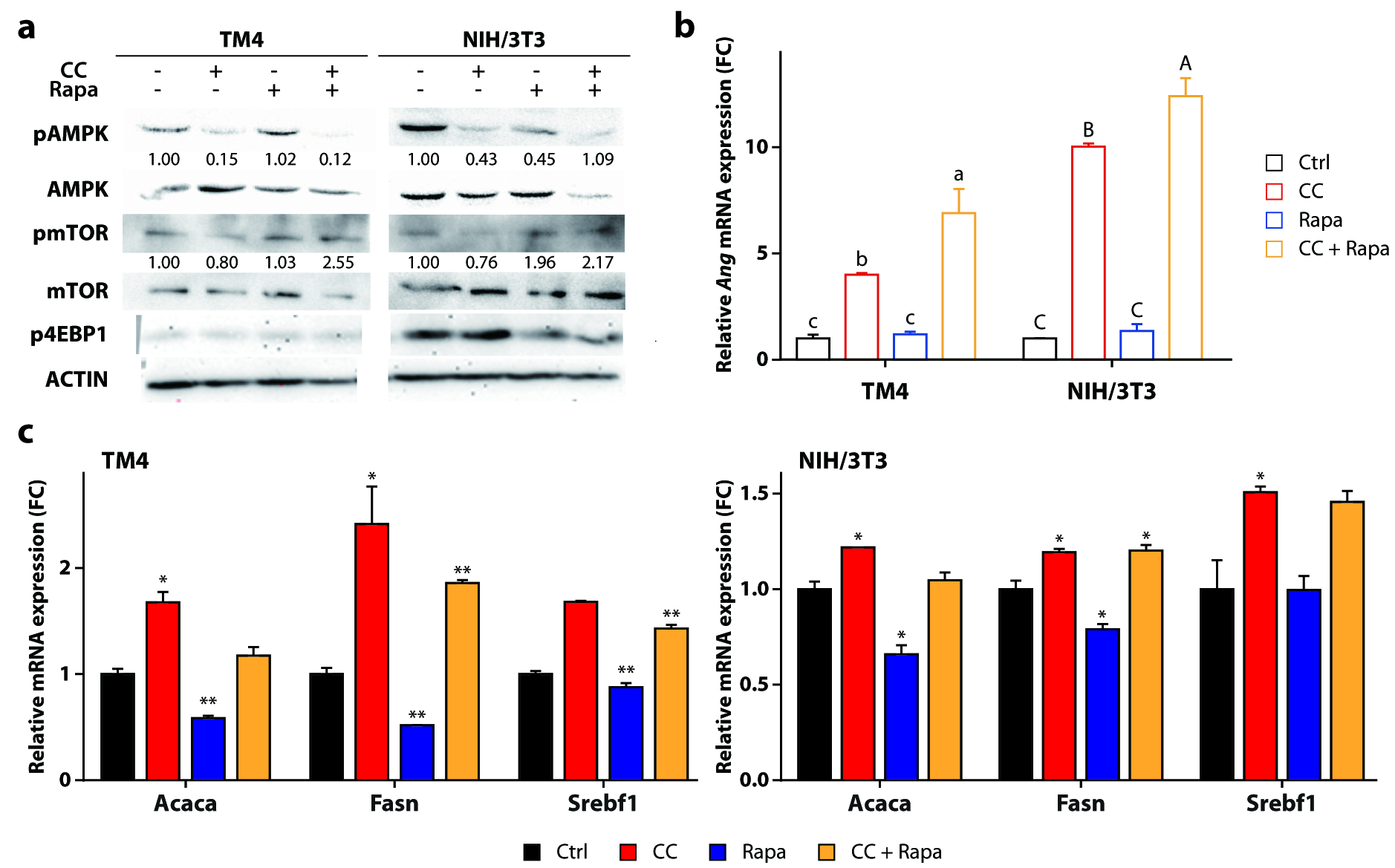
**

**Supplementary Figure 3. AMPK inhibition-mediated Angiogenin induction independent of mTOR.**

(a) Western blot results of proteins related with AMPK-mTOR pathways in TM4 and NIH/3T3 cell lines using compound C (CC), rapamycin (Rapa), and co-treatment. The number represents the densiometric comparison of the expression of pAMPK and pmTOR proteins against AMPK and mTOR, respectively. (b) Angiogenin gene expression. Gene expression was quantified after 12 hours of treatment. mRNA expression level was normalized to *Actin*. Data are represented as mean ± standard deviation. Statistical analysis was performed using One-way ANOVA with Tukey post-hoc test. Different letters are significantly different. (C) lipid metabolism-related gene expression after treatment. Relative levels of mRNA expression were calculated by normalization against *Actin* expression. Data are represented as mean ± standard deviation. Statistical analysis was performed using the student’s t-test. * p<0.05 and **, p<0.05 vs Ctrl.
